# Unfolding Mechanism and Fibril Formation Propensity of Human Prion Protein in the Presence of Molecular Crowding Agents

**DOI:** 10.3390/ijms25189916

**Published:** 2024-09-13

**Authors:** Manoj Madheswaran, Nataliia Ventserova, Gianluca D’Abrosca, Giulia Salzano, Luigi Celauro, Federico Angelo Cazzaniga, Carla Isernia, Gaetano Malgieri, Fabio Moda, Luigi Russo, Giuseppe Legname, Roberto Fattorusso

**Affiliations:** 1 Department of Environmental, Biological and Pharmaceutical Sciences and Technologies (DISTABiF), Università degli Studi della Campania Luigi Vanvitelli, 81100 Caserta, Italy; 2 Department of Clinical and Experimental Medicine, Università degli Studi di Foggia, 71122 Foggia, Italy; 3 Department of Neuroscience, Scuola Internazionale Superiore di Studi Avanzati, 34136 Trieste, Italy; 4 Division of Neurology 5–Neuropathology, Fondazione IRCCS Istituto Neurologico Carlo Besta, 20133 Milan, Italy; 5 SSD Laboratory Medicine, Fondazione IRCCS Istituto Neurologico Carlo Besta, 20133 Milan, Italy

**Keywords:** prion protein, thermal unfolding, molecular crowding, Ficoll, amyloid fibrils

## Abstract

The pathological process of prion diseases implicates that the normal physiological cellular prion protein (PrP^C^) converts into misfolded abnormal scrapie prion (PrP^Sc^) through post-translational modifications that increase β-sheet conformation. We recently demonstrated that HuPrP(90–231) thermal unfolding is partially irreversible and characterized by an intermediate state (β-PrPI), which has been revealed to be involved in the initial stages of PrP^C^ fibrillation, with a seeding activity comparable to that of human infectious prions. In this study, we report the thermal unfolding characterization, in cell-mimicking conditions, of the truncated (HuPrP(90–231)) and full-length (HuPrP(23–231)) human prion protein by means of CD and NMR spectroscopy, revealing that HuPrP(90–231) thermal unfolding is characterized by two successive transitions, as in buffer solution. The amyloidogenic propensity of HuPrP(90–231) under crowded conditions has also been investigated. Our findings show that although the prion intermediate, structurally very similar to β-PrPI, forms at a lower temperature compared to when it is dissolved in buffer solution, in cell-mimicking conditions, the formation of prion fibrils requires a longer incubation time, outlining how molecular crowding influences both the equilibrium states of PrP and its kinetic pathways of folding and aggregation.

## 1. Introduction

Prion diseases, also known as transmissible spongiform encephalopathies (TSEs), are rare incurable neurological diseases with an unusual disease etiology that affect both humans and animals [1,2]. TSEs include the Creutzfeldt-Jakob disease (CJD), Gerstmann–Sträussler–Scheinker syndrome (GSS), fatal familial insomnia (FFI) and Kuru in humans, scrapie in goats and sheep, bovine spongiform encephalopathy (BSE) or ‘mad cow disease’ in cattle, transmissible mink encephalopathy (TME) in minks, feline spongiform encephalopathy (FSE) in camels and cats, and chronic wasting disease (CWD) in cervids [3,4,5,6,7]. The pathological process of this disease implicates that the normal physiological cellular prion protein (PrP^C^) converts into a misfolded abnormal scrapie prion (PrP^Sc^) through post-translational modifications that lead to an increased β-sheet conformation [8]. This conversion leads to the accumulation of amyloid fibrils in the brain, causing neurodegeneration [9]. The human prion protein (HuPrP) gene is encoded by the *PRNP* gene, which is highly conserved among species, and its complete open reading frame is invariably located within a single exon [8,10,11]. In humans, prion diseases are classified into three categories: sporadic, genetic, and acquired. The majority of cases reported in humans are sporadic [12]. Although the origin of sporadic Creutzfeldt-Jakob disease (sCJD) is still unclear, it has been suggested that a somatic *PRNP* mutation or the spontaneous conversion of PrP^C^ to PrP^Sc^ could be the reason [12]. Genetic prion diseases are classified based on clinical symptoms and neuropathological attributes and consist of genetic CJD (gCJD), fatal familial insomnia (FFI), and Gerstmann-Sträussler-Scheinker (GSS) disease. The mutations in *PRNP* are autosomal dominant, highly penetrant, and consist of missense mutations, insertions, and deletions, usually provoking disease after 55 years [13]. Acquired prion diseases have been transmitted between individuals (kuru and iatrogenic CJD) and, in rare cases, from cattle to humans (a variant of Creutzfeldt-Jakob disease (vCJD)) [14,15].

PrP^C^ is a cell surface glycoprotein found in the central nervous system (CNS) [8]. Recent studies suggest that PrP^C^ is involved in various cellular processes, such as neuronal growth [16], cell signaling [17,18], role against stress [19,20], cell adhesion [21], synapse formation, sleep patterns, and metal ion homeostasis [22]. The exact function of PrP^C^ is still unknown. Human prion protein encodes 253 amino acid residues in its mature form; the first 22 residues are cleaved after translation, and the last 23 residues are cleaved prior to translation. In general, HuPrP comprised of 209 residues (23–231). The protein is attached to the outer surface of cellular membranes by a glycosylphosphatidylinositol anchor at its C terminus [23]. Nuclear magnetic resonance (NMR) studies revealed that PrP^C^ has an intrinsically disordered N-terminal region (23–126) and structured C-terminal region (127–231) [24,25,26]. The structured C-terminal region is primarily α-helical, with three helices (α1, α2, and α3) and a short antiparallel β-sheet (β1–β2) [27,28]. The bulk of the globular domain is formed by the helices α2 and α3, which are covalently bridged by a disulfide bond between Cys179 and Cys214, to which the β-sheet and α1 are anchored [29]. The intrinsically disordered N-terminal region (residues 23–126) contains two charged clusters (CC1, residues 24–30, and CC2, residues 101–110), the octarepeat region (OR, residues 59–90), the non-octarepeat region (non-OR, residues 91–110) and a hydrophobic domain (HD) (residues 111–126). Folding intermediates have been detected in kinetic studies of HuPrP at pH 5.5, as well as of pathogenic variants of ovine PrP, but not in similar studies conducted on mouse PrP at pH 7.0 [30,31,32]. We recently demonstrated that HuPrP(90–231) thermal unfolding is partially irreversible and characterized by an intermediate state (β-PrPI) [33], whose detailed structural description of HuPrP conformation equilibria has been obtained at pH = 5.5 [33]. In this study, the N-terminal domain has been shown to play a key role in the thermodynamic stability of the protein since its absence induces a more complex thermal unfolding, particularly at pH 5.5 [33]. Furthermore, we have also demonstrated that the native state of HuPrP(90–231) at room temperature is in conformational equilibrium with a low-populated state that presents structural similarity to β-PrPI. Importantly, β-PrPI has been revealed to be involved in the initial stages of PrP^C^ fibrillation, having a seeding activity comparable to human infectious prions.

Among the factors that can affect the conformational equilibria of the prion protein, high concentrations of macromolecules characterizing the cellular environment may play a vital role in modulating the folding mechanism of the prion protein, favoring or disfavoring the formation of stable misfolded intermediate states [34]. As an example, a recent study showed the effect of different organic solvents (acetone, acetonitrile, ethanol, and tetrahydrofuran) on the SDS-induced aggregation of lysozyme. In particular, the presence of acetone impeded SDS-induced lysozyme aggregation into amyloid fibrils from denatured protein in a concentration-dependent manner [35]. Based on these considerations, here, we report the thermal unfolding characterization, in cell-mimicking conditions, of the truncated (HuPrP(90–231)) and full-length (HuPrP(23–231)) human prion protein by CD and NMR spectroscopy. Yet, we also investigated the amyloidogenic propensity of HuPrP(90–231) under crowded conditions. Our findings revealed that although the β-PrPI forms at a lower temperature compared to when it is dissolved in a buffer solution, the formation of prion fibrils requires a longer incubation time.

## 2. Results

### 2.1. CD Spectroscopy

**Figure 1:**
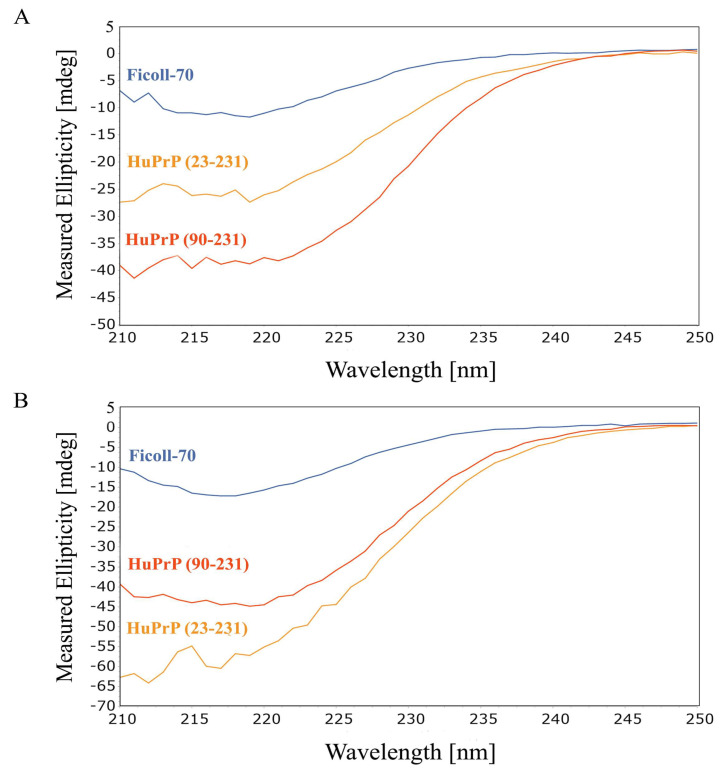
(**A**) Far-UV CD spectra of HuPrP(23–231) and HuPrP(90–231) at pH 5.5. Experiments were carried out in presence of Ficoll-70 (150 g/L) at 25 °C (Blue: Ficoll-70, yellow: HuPrP(23–231), orange: HuPrP(90–231)). (**B**) Far-UV CD spectra of HuPrP(23–231) and HuPrP(90–231) at pH 6.8. Experiments were carried out in in presence of Ficoll-70 (150 g/L) at 25 °C (Blue: Ficoll-70 curve, orange: HuPrP(90–231), yellow: HuPrP(23–231)).

CD spectroscopy has been initially utilized to investigate, at low resolution, the thermal unfolding of HuPrP(90–231) in the presence of Ficoll-70 at the concentration of 150 g/L. The spectra of HuPrP(90–231) and HuPrP(23–231) in the presence of Ficoll-70 at 25 °C and at pH = 5.5 and at pH = 6.8 are shown in Figure 1A and 1B, respectively. Overall, CD data clearly indicate that the presence of the crowders does not alter the secondary structure of both HuPrP(90–231) and HuPrP(23–231).

**Figure 2:**
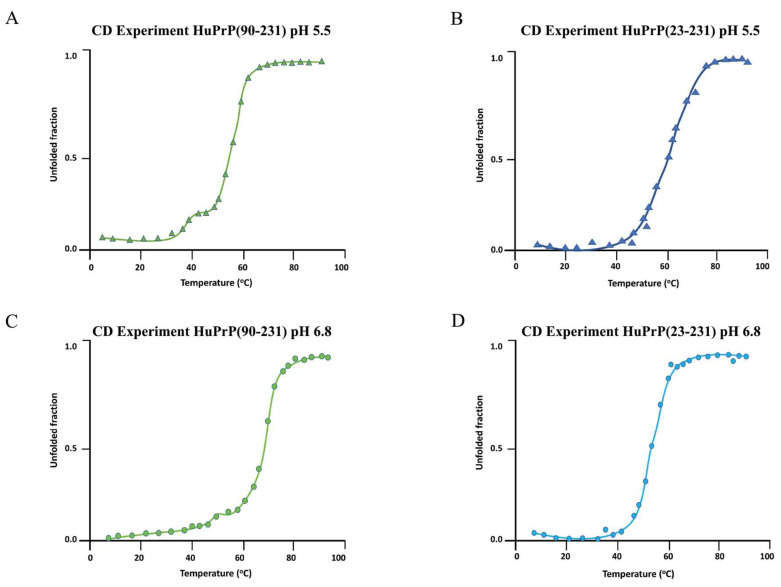
CD thermal unfolding of HuPrP proteins in the presence of Ficoll-70. Thermal melt plots are a function of temperature. Measurements were performed at pH 5.5 and 6.8 at different temperatures ranging from 5 °C to 90 °C. Data were fitted according to two- and three-state models. HuPrP(90–231) at pH 5.5 (**A**), HuPrP(23–231) at pH 5.5 (**B**), HuPrP(90–231) at pH 6.8 (**C**), and HuPrP(23–231) at pH 6.8 (**D**).

After that, we performed CD thermal unfolding experiments on HuPrP(23–231) and HuPrP(90–231) (Figure 2A–D) upon the addition of Ficoll-70 at neutral and mildly acidic pH. Full-length HuPrP(23–231) exhibits a two-state cooperative thermal unfolding with a midpoint transition temperature (T_m_) of 59 ± 3 °C at pH 5.5 and 53 ± 4 °C at pH 6.8, respectively (Table 1). At pH 5.5, the truncated HuPrP(90–231) exhibits a more complex thermal unfolding curve that results in two successive transitions, the first with a T_m_ of 41 ± 2 °C and the second with a T_m_ of 54 ± 2 °C (Table 1). At pH 6.8, HuPrP(90–231) unfolding shows two closer transitions, the first at 50 ± 2 °C and the second at 66 ± 2 °C. Interestingly, the comparison of the T_m_s estimated for HuPrP(23–231) and HuPrP(90–231) in cell-mimicking conditions indicates that, as previously reported in dilute buffer solution, the N-terminal domain plays an important role in the thermodynamic stability of the HuPrP protein. Its absence induces a three-state thermal unfolding process, forming a stable intermediate state, particularly at pH 5.5.

Nevertheless, the molecular crowding significantly reduces the T_m_s of the truncated HuPrP(90–231) and full-length HuPrP(23–231) at both pHs (see Table 1).

### 2.2. NMR Structural Investigation of HuPrP(90–231) in the Presence of Ficoll-70

In order to explore the crowding effects on the overall structural architecture of truncated HuPrP, we performed a high-resolution NMR structural characterization of HuPrP(90–231) at 25 °C in the presence of 150 and 50 g/L of Ficoll-70. At both polymer concentrations, in the ^1^H-^15^N HSQC of HuPrP(90–231), there is a good dispersion of resonances in both proton and nitrogen dimensions, indicating that the presence of the crowding agent does not destabilize the native form of the protein.

**Figure 3:**
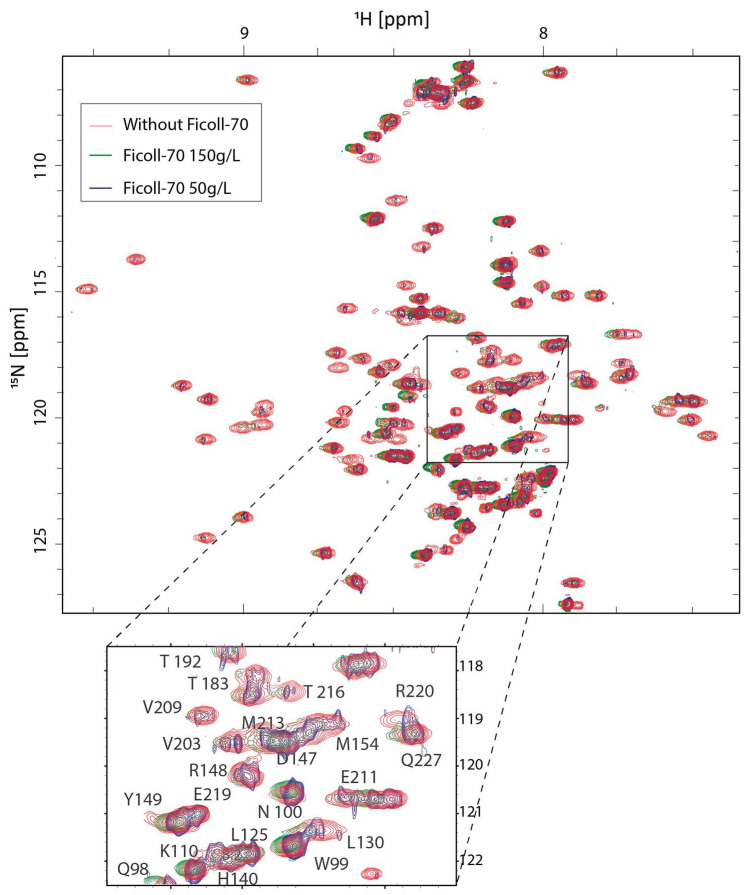
^1^H-^15^N HSQC spectra for HuPrP(90–231). Overlay of ^1^H-^15^N HSQC spectra of HuPrP(90–231) acquired in dilute buffer (red), in the presence of 150 g/L of Ficoll-70 (green), and in the presence of 50 g/L (blue) of Ficoll-70 acquired on 600 MHz spectrometer at 25 °C and pH 5.5.

**Figure 4:**
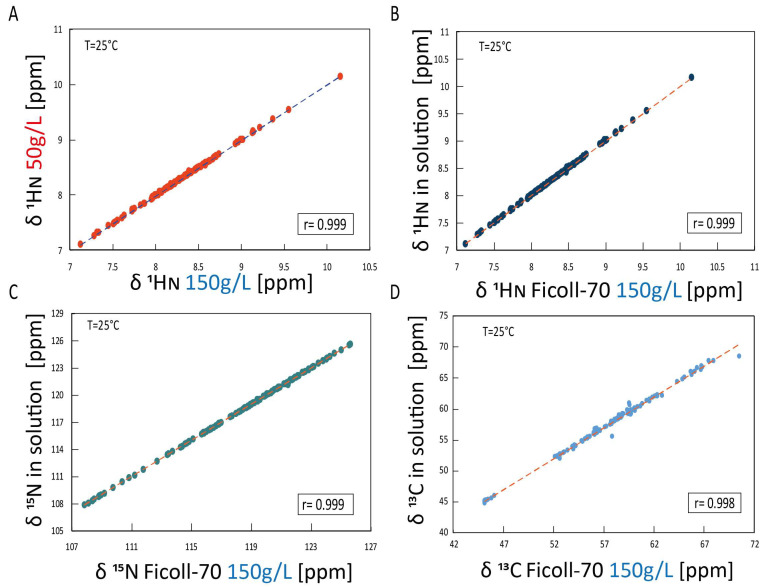
Chemical shift correlation plots in the different conditions (**A**) The ^1^H_N_ chemical shift correlation plot of HuPrP(90–231) in two different concentrations of Ficoll-70 (50 g/L against 150 g/L). (**B**) The ^1^H_N_ chemical shift correlation plot of HuPrP(90–231) in solution against Ficoll-70 150 g/L. (**C**) The ^15^N chemical shift correlation plot of HuPrP(90–231) in solution against Ficoll-70 150 g/L. (**D**) The ^13^Cα chemical shift correlation plot of HuPrP(90–231) in solution against Ficoll-70 150 g/L.

Figure 3 reports the two-dimensional ^1^H-^15^N HSQC HuPrP(90–231) spectrum, acquired either at 150 and 50 g/L Ficoll-70 concentrations, superimposed to the ^1^H-^15^N HSQC HuPrP(90–231) spectrum recorded in dilute buffer solution. The chemical shift correlation plots (Figure 4A–C) also show that only small changes in the ^15^N and ^1^H chemical shifts are observed in the three different conditions, indicating that the three-dimensional organization of HuPrP(90–231) observed in solution is conserved in both cell-mimicking conditions. These findings were further confirmed by the evaluation of Cα chemical shifts that are sensitive probes for secondary structure. As reported in Figure 4D, the comparison of solution Cα of HuPrP(90–231) with the values observed in the presence of the polymer confirms that HuPrP(90–231) secondary structure is not significantly affected by the presence of a crowded environment.

### 2.3. NMR Thermal Unfolding Characterization

**Figure 5:**
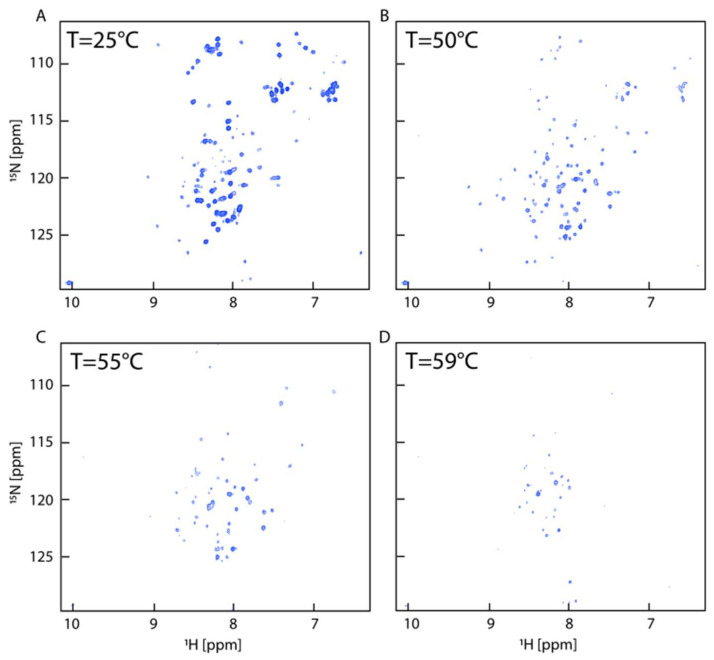
NMR thermal unfolding. (**A**–**D**) ^1^H-^15^N HSQCs were used to monitor the thermal unfolding of HuPrP(90–231) in the presence of 150 g/L Ficoll-70 acquired at pH 5.5 on a 600 MHz spectrometer equipped with a cryoprobe.

To investigate, at atomic resolution, the complex folding mechanism of HuPrP(90–231) shown by the CD analysis, we carried out an NMR characterization of HuPrP(90–231) thermal unfolding dissolved in a Ficoll-70 concentration of 150 g/L at pH 5.5, by acquiring a series of two-dimensional ^1^H-^15^N HSQCs between 5 °C and 80 °C at intervals of 5 °C and at 2 °C intervals in the range 55–75 °C (Figure 5). Most residues started to disappear at around 55 °C (Figure 4C). A well-preserved spectral dispersion is retained at 50 °C (Figure 5B), indicating the presence of a partially folded structure at this temperature.

**Figure 6:**
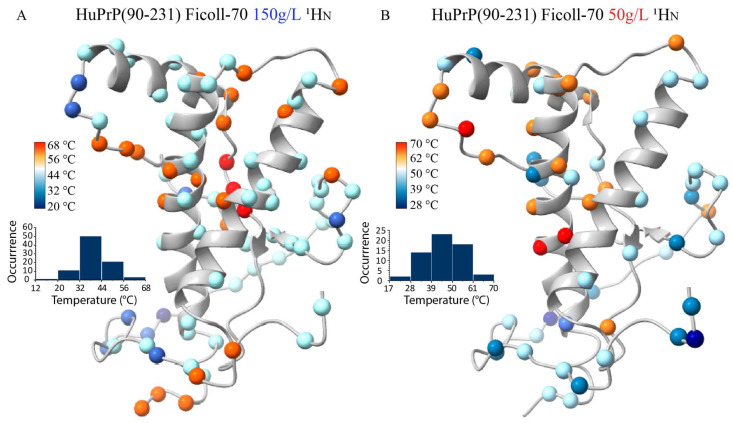
“Atom-by-atom” unfolding behavior of HuPrP(90–231). Ribbon drawing representation of the HuPrP(90–231) in both Ficoll-70 concentrations: (**A**) 150 g/L, (**B**) 50 g/L. NMR structure showing the thermal stability estimated from the ^1^H_N_ chemical shift variations mapped on their corresponding atoms. The color displayed for each atom corresponds to the T_m_. Balls with similar colors have similar T_m_s. The insets show the distribution of melting temperature.

We estimated the midpoint temperatures (T_m_s) of the first thermal transition for most atoms by evaluating ^1^H_N_ chemical shift variations as a function of temperature. T_m_s resulting from this analysis have been mapped onto the HuPrP(90–231) NMR structure (Figure 6A), revealing that all the analyzed atoms experience a structural transition with a proper T_m_s within the 20–68 °C range. Nicely reconciling with the CD data, T_m_s mapping shows a clear distributional behavior of the structural transition centered at 39 °C.

**Figure 7:**
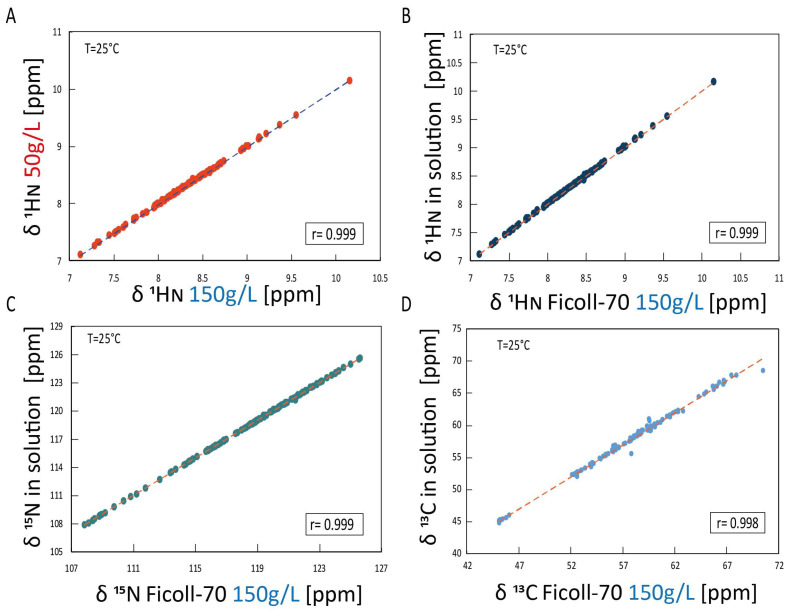
The intermediate chemical shift and secondary chemical shift comparison in different environments. (**A**) ^1^H_N_ and (**B**) ^15^N chemical shift correlation plot of HuPrP(90–231) in Ficoll-70 50 g/L obtained at 50 °C against HuPrP(90–231) in dilute solution obtained at 61 °C [33]. (**C**) ^1^H_N_ and (**D**) ^15^N chemical shift correlation plot of HuPrP(90–231) in Ficoll-70 150 g/L obtained at 50 °C against HuPrP(90–231) in dilute solution obtained at 61 °C [33].

Interestingly, a similar analysis has also been carried out on the truncated HuPrP(90–231) dissolved in 50 g/L Ficoll-70, giving very similar results (Figure 6B), showing that differences in the concentration of the crowding agent do not appear to result in different thermal unfolding processes. In both conditions, as a matter of fact, the thermal transitions lead to the formation of a conformational intermediate state that may have structural similarities with the β-PrPI detected in the solution. To fully address this latter point, we compared ^1^H and ^15^N chemical shifts measured at 50 °C for the truncated HuPrP(90–231) dissolved either in 150 g/L and 50 g/L Ficoll-70 concentrations with the shifts reported for β-PrPI identified in buffer solution at 61 °C (Figure 7A–D).

The correlation plots show that the intermediate forming at 50 °C, either at the lower or higher Ficoll-70 concentrations, have very similar ^1^H and ^15^N chemical shifts and are, therefore, structurally comparable.

### 2.4. ThT Fibrillation Experiments

**Figure 8:**
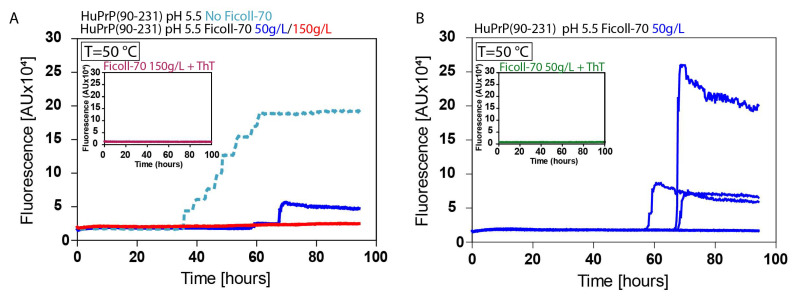
Role of the β-PrPI intermediate state in amyloid fibril formation and in prion protein conversion in different environments. (**A**) Average ThT fluorescence intensity was plotted against time (T = 50 °C) for HuPrP(90–231): in dilute solution (light blue dotted line), Ficoll-70 50 g/L concentration (dark blue line), Ficoll-70 at 150 g/L (red line). Insert reports the control experiment containing ThT + Ficoll-70 150 g/L. (**B**) Aggregation of the reaction substrate: HuPrP(90–231) with a longer lag-phase and subsequent self-assembly. Insert reports the control experiment containing ThT + Ficoll-70 50 g/L.

To evaluate the amyloidogenic propensity of the truncated HuPrP(90–231), we performed in vitro fibrillation experiments using ThT fluorescent dye (Figure 8). The two samples, dissolved in 150 g/L and 50 g/L Ficoll-70 solutions, were incubated at 50 °C at pH 5.5, and aggregations were performed by subjecting the samples to several cycles of incubation and shaking. Fibril growth was monitored using ThT in real-time. Our results show that the aggregation of HuPrP(90–231) is significantly faster in 50 g/L Ficoll-70 concentrations, though slower than in dilute buffer solution. Particularly, fibrillation is observed in three out of eight wells after 50 h, whereas at a concentration of 150 g/L, fibrillation occurs in two out of eight wells but with a much lower absorbance signal. Furthermore, no fibrillation is observed at room temperature, as it happens in buffer solution. Overall, NMR and ThT data demonstrate that the concentration of the molecular crowding plays a crucial role in modulating the kinetic of the HuPrP(90–231) aggregation reaction by which the formation of amyloid aggregates is driven by a misfolded state having structural similarity with β-PrPI.

## 3. Discussion

Since the first structural characterizations of PrP^C^ have been published [36], a significant number of computational and experimental studies have been performed to shed light on the first stages of protein misfolding leading to the PrP^C^-PrP^Sc^ transition [37,38,39,40]. In most experimental studies, non-native conditions induced by lowering pH or adding chemical denaturants have been exploited to investigate partially unfolded forms of native or pathogenically mutated PrP^C^ in fibril formation [31,41].

Thus, the study of the conformational equilibria and unfolding/folding processes of PrP^C^ under different conditions represents a critical point to investigate the molecular basis of the PrP^C^ to PrP^Sc^ transition. In this context, molecular crowding, reproducing the high concentration of macromolecules within the physiological cellular environment, is known to affect thermodynamics and kinetics of biochemical processes [42,43]. Recently, via nuclear magnetic resonance (NMR) methodologies, we investigated at the atomic level the mechanism of human HuPrP(90–231) thermal unfolding in dilute buffer conditions, characterizing the conformational equilibrium between its native structure and a β-enriched intermediate state, named β-PrPI [33].

In this study, we analyzed the structure and the thermal stability of HuPrP in solutions containing 50 g/L and 150 g/L of Ficoll-70 to mimic the crowded physiological cellular environment.

Thermal unfolding analysis of human PrP^C^ has been performed at different pHs on the full-length human HuPrP(23–231) and the truncated HuPrP(90–231) to identify conformational equilibria characterizing PrP^C^ unfolding processes in these new conditions.

Our characterization was initially performed by CD analysis of the two constructs in Ficoll-70 (protein concentration 30 μM, Ficoll-70 150 g/L), showing that the presence of the crowders did not significantly alter the overall structure of the prion protein (Figure 3 and Figure 4). Next, a thermal unfolding analysis of human PrP^C^ has been performed to identify the conformational equilibria characterizing PrP^C^ unfolding processes. The CD analysis shows that, similarly to what was observed in dilute buffer solution, the presence of the N-terminal tail, constituted by residue 23–89, influences the mechanism of human PrP^C^ thermal unfolding.

In the presence of the molecular crowding agent, HuPrP(23–231) is not characterized by a thermal-induced intermediate and shows thermodynamic properties completely different from those observed for HuPrP(90–231).

As a matter of fact, full-length HuPrP(23–231) thermally unfolds following a cooperative two-state mechanism at pH = 6.8 and pH = 5.5, even though with different T_m_s (52 ± 4 °C and 59 ± 3, respectively). On the other hand, HuPrP(90–231) thermal unfolding has a more complex behavior, characterized by two successive transitions. In particular, at pH 6.8, the midpoint temperatures (T_m_s) of the two transitions are 50 ± 2 °C and 66 ± 2 °C, while at pH 5.5, they separate at 41 ± 2 °C and 54 ± 2 °C.

Overall, the presence of the crowding agents appears to destabilize the protein thermally.

The thermal destabilization of HuPrP(90–231) in Ficoll-70, as evidenced by CD data, indicates that the protein is more prone to partial thermal unfolding under these conditions, which could be an initial step toward misfolding and aggregation.

Thus, our analysis has successively focused on HuPrP(90–231) at pH 5.5. The ^1^H-^15^N HSQC acquired at room temperature and at increasing temperatures (protein concentration 30 μM, Ficoll-70 150 g/L) has substantially confirmed the CD results. The ^1^H-^15^N HSQC at 25 °C shows that all resonances experience only minor chemical shift differences with respect to the same spectrum recorded in dilute buffer conditions. The ^1^H-^15^N HSQC for HuPrP(90–231) at pH 5.5 recorded at 50 °C indicates the presence of an intermediate conformation. Overall, the backbone proton T_m_s (centered at 39 °C) derived by NMR analysis well corresponds to the first thermally induced unfolding transition described by our CD results, leading to the partial unfolding of the globular domain. The same NMR experiments have been conducted at a different Ficoll-70 concentration to explore the effect of Ficoll-70 concentration on the protein conformational equilibria. NMR experiments conducted at pH 5.5 with Ficoll-70 at a concentration of 50 g/L showed results similar to those at 150 g/L, as demonstrated by correlation plots. In thermal denaturation experiments, the melting temperature was found to be 45.5 °C, indicating that the decrease of Ficoll-70 concentration positively affects the stability of the protein.

The correlation plots between the chemical shifts of the resonances at 50 °C in Ficoll-70 at 150 g/L and at 50 g/L and 61 °C in dilute buffer solution indicate that the intermediate formed at high temperature is similar in the three different conditions. In buffer solution at 61 °C, the most significant chemical shift perturbations affect nuclei involved in all the secondary structure elements. A β-structure extension is observed that is coupled with a loss of helical content of the three native helices, particularly within the contact surfaces of α2 and α3, which include a disulfide bridge, defining the intermediate state β-PrPI involved in the initial stage of PrPC fibrillation. Therefore, given the structural similarities between β-PrPI and the intermediate states detected for HuPrP(90–231) in the presence of Ficoll-70, we investigated the amyloidogenic properties of the misfolded states observed within cell-mimicking physiological conditions.

Therefore, we performed ThT experiments at pH 5.5 in two different conditions: 50 g/L and 150 g/L of Ficoll-70. At 50 °C, we observe faster fibril formation at 50 g/L of Ficoll70 with respect to 150 g/L of Ficoll-70.

This observation can be attributed to several factors related to molecular crowding and its effects on protein folding and aggregation kinetics.

Molecular crowding generally favors the compact, native state of proteins by excluding the volume available for unfolding [44]. However, at intermediate crowding levels (50 g/L), although with a slower kinetic with respect to the dilute buffer solution, there may be sufficient space for partially unfolded intermediates to form and interact, promoting fibril formation. At higher concentrations of Ficoll-70 (150 g/L), the higher viscosity and reduced free volume significantly impede the diffusion of prion monomers and oligomers, slowing down the rate at which they can form fibrils.

The initial phase of fibril formation involves the aggregation of partially unfolded prion proteins. At lower Ficoll-70 concentrations (50 g/L), the crowding effect may allow interactions between these misfolded conformational states. On the other hand, higher concentrations of Ficoll-70 (150 g/L) significantly slow down this elongation phase due to steric hindrance and slower diffusion rates.

Nonetheless, at higher concentrations, non-amyloid aggregates that do not contribute to ThT-detectable fibrils could also form [45].

## 4. Materials and Methods

### 4.1. Materials

The HuPrP(23–231) and HuPrP(90–231) were expressed and purified as previously reported [33].

Ficoll PM70 (average molecular mass 70 KDa) powder was purchased from Sigma–Aldrich and stored at room temperature. Sodium acetate buffer (pH 5.5) and sodium phosphate buffer (pH 6.8) were prepared and stored at 4 °C. Pre-weighed amounts of Ficoll PM70 powder were dissolved in sodium acetate buffer (pH 5.5) and sodium phosphate buffer (pH 7.0) at room temperature with gentle stirring.

### 4.2. CD Spectroscopy

CD samples contained HuPrP(23–231) or HuPrP(90–231) in 300 μL of 20 mM sodium acetate at pH 5.5 or sodium phosphate buffer at pH 6.8 with the same ionic strengths. The Ficoll-70 concentration in each sample was 150 g/L, while the protein concentration was 30 μM. The thermal denaturation of prion protein samples was assessed using a JASCO J-815 CD spectropolarimeter equipped with Peltier temperature control. CD spectra were recorded at 5 °C intervals from 5 to 35 °C and at 3 °C intervals from 35 to 90 °C. After that, all samples were cooled to 25 °C for a final set of spectra measurements. All data were collected in triplicate with a bandwidth of 1 nm and a scanning speed of 50 nm/min and were normalized against reference spectra to eliminate the background contribution of the buffer and of the crowding agent. The data were analyzed using a two- and three-state folding model. The errors reported are derived from the fitting procedures. Control experiments were conducted with the Ficoll-70 (150 g/L) solution to evaluate the behavior of the blank solutions upon temperature increase.

### 4.3. NMR Experiments

NMR experiments were conducted at 25 °C using a Bruker AVIII HD 600 MHz spectrometer equipped with a triple resonance Prodigy N2 cryoprobe featuring a *z*-axis pulse field gradient. NMR samples for the chemical shift assignment of ^15^N-^13^C labeled HuPrP(23–231) and ^15^N-^13^C labeled HuPrP(90–231) proteins were prepared at a concentration of 80 μM in a solvent mixture of 90% H_2_O/10% D_2_O, and 150 and 50 g/L Ficoll-70 dissolved in sodium acetate buffer at pH 5.5. Two-dimensional ^1^H-^15^N HSQC and ^1^H-^13^C HSQC spectra were acquired for both HuPrP(23–231) and HuPrP(90–231). Backbone resonances Cα, N, HN, and Hα of HuPrP(90–231) were assigned by analyzing standard triple resonance experiments [46], including 3D HNCA, 3D CBCA(CO)NH, and 3D HNHA, using the deposited chemical shifts under the BMRB accession codes 4402, 18426, and 17780. The chemical shift assignment of HuPrP(90–231) protein at different temperatures was performed by tracking peak trajectories over the temperature range. Spectra were processed using NMRpipe [47] and analyzed with SPARKY [48] and CARA [49]. The ^1^H, ^13^C, and ^15^N chemical shifts were indirectly calibrated using external DSS references.

The quality of the NMR samples was checked over time. In all investigated conditions, the quality of the samples and of the corresponding NMR spectra allow us to exclude the presence of protein liquid-liquid phase separation systems [50,51].

### 4.4. NMR Chemical Shifts and Thermal Analysis

^1^H-^15^N and ^1^H-^13^C HSQC NMR thermal experiments for HuPrP(90–231) were collected every 5 °C from 5 °C to 55 °C and every 3 °C from 55 °C to 90 °C. All NMR spectra used for the thermal unfolding characterization were indirectly referenced using external DSS references. 2D ^1^H-^15^N HSQC experiments were acquired with 32 scans per t1 increment, a spectral width of 1459.43 Hz along t1 and 7211.54 Hz along t2, 2048 × 256 complex points in t2 and t1, respectively, and a 1.0 s relaxation delay. Spectra were apodized with a square cosine window function and zero-filled to a matrix size of 4096 x 4096 before the Fourier transform and baseline correction. ^1^H chemical shifts at different temperatures were externally referenced to DSS, while ^15^N and ^13^C shifts were indirectly calibrated to DSS. Individual fits of HN, Cα, and Hα chemical shifts as a function of temperature were performed with two-state transitions. The Pearson correlation was used to estimate the correlation between the ^1^H_N_ and ^15^N chemical shifts observed for HuPrP(90–231) in the presence of Ficoll-70 (150 g/L and 50 g/L) with those measured in an acetate buffer solution (pH 5.5). The CHIMERA 1.18 software was utilized to evaluate and visualize the data [52].

### 4.5. Aggregation Assay

Human truncated recombinant PrP protein (HuPrP(90–231)) was filtered through a 100 kDa Nanosep centrifugal device (Pall Corporation, New York, NY, USA). For the aggregation assay, we have prepared three reaction mixes containing different concentrations of Ficoll-70: (1) 0 g/L, (2) 50 g/L, and (3) 150 g/L. Each reaction mix (100 µL final volume) was composed of 10 mM PBS (pH 7.4), acetate buffer solution (pH 5.5), 150 mM NaCl, 0.135 mg/mL HuPrP(90–231), 1 mM EDTA, 0.002% SDS, 10 μM thioflavin T (ThT), and Ficoll-70 at the appropriate concentration (0, 50 or 150 g/L). Eight replicates for each condition were analyzed in a black 96-well optical flat bottom plate (Thermo Scientific, Waltham, MA, USA). The plate was sealed with a sealing film (Thermo Scientific) and inserted into a FLUOstar OMEGA microplate reader (BMG Labtech, Ortenberg, Germany). The plate was subjected to alternating cycles of shaking (1 min at 600 rpm, double orbital) and incubation (1 min at 50 °C). The mean fluorescence values (expressed as arbitrary units, AU) of the aggregating replicates were plotted in a graph against time.

## 5. Conclusions

In this study, we report the thermal unfolding characterization, in cell-mimicking conditions, of the truncated (HuPrP(90–231)) and full-length (HuPrP(23–231)) human prion protein by means of CD and NMR spectroscopy.

On the one hand, our results show that prion proteins appear to be thermally destabilized in the crowded environment generated by exploiting Ficoll-70. Particularly, significant concentrations of HuPrP(90–231) intermediate are already formed at the temperature of the human body, 37 °C, indicating that the increase of molecular crowding at physiological temperature stabilizes the prion intermediate shown to be involved in prion fibrillation [53]. On the other hand, ThT analysis indicates that the presence of the crowding agent slows down the formation of amyloid fibers, outlining how crowding influences both the equilibrium states of PrP and its kinetic pathways of folding and aggregation.

These findings suggest that the different degrees of macromolecular crowding within the intracellular environment could influence the kinetics of prion protein misfolding and aggregation differently. Less crowded cellular regions may promote a faster process of fibril formation, while more crowded ones may slow it down.

Our findings highlight the intricate balance between protein stability, molecular interactions, and molecular crowding effects, providing a further piece in the complex puzzle of prion disease mechanisms.

## Figures and Tables

**Table 1:** Thermal stability of prion protein samples in the presence of Ficoll-70 at different pH values estimated via CD. Midpoint transition temperatures (T_m_) (°C) of truncated HuPrP(90–231) and HuPrP(23–231) unfolding processes in the presence of the macromolecular crowding agent experiments at pH values 5.5 and pH 6.8 and compared with CD data acquired in dilute buffer conditions, indicated by “*” [33].

*Protein*	*Condition*	*T_m_*_1_ (°C)	*T_m_*_2_ (°C)
*HuPrP(90–231)*	Ficoll-70 pH 5.5	41 ± 2	54 ± 2
	Buffer solution pH 5.5	55 ± 1 *	72 ± 1 *
*HuPrP(23–231)*	Ficoll-70 pH 5.5	-	59 ± 3
	Buffer solution pH 5.5	-	69 ± 3 *
*HuPrP(90–231)*	Ficoll-70 pH 6.8	50 ± 2	66 ± 2
	Buffer solution pH 6.8	59 ± 3 *	68 ± 2 *
*HuPrP(23–231)*	Ficoll-70 pH 6.8	-	53 ± 4
	Buffer solution pH 6.8	-	59 ± 1 *

## Data Availability

Data contained within the article.
